# Supplementary material for: Beyond commensalism: genomic insights into micrococcin P1-producing Staphylococcus chromogenes
Source: mSphere. 2025 Nov 24;10(12):e00733-25. doi: 10.1128/msphere.00733-25 (PMC12724294; doi:10.1128/msphere.00733-25)
Supplement: Supplemental Figures — Figures S1 to S8. [file msphere.00733-25-s0002.docx]

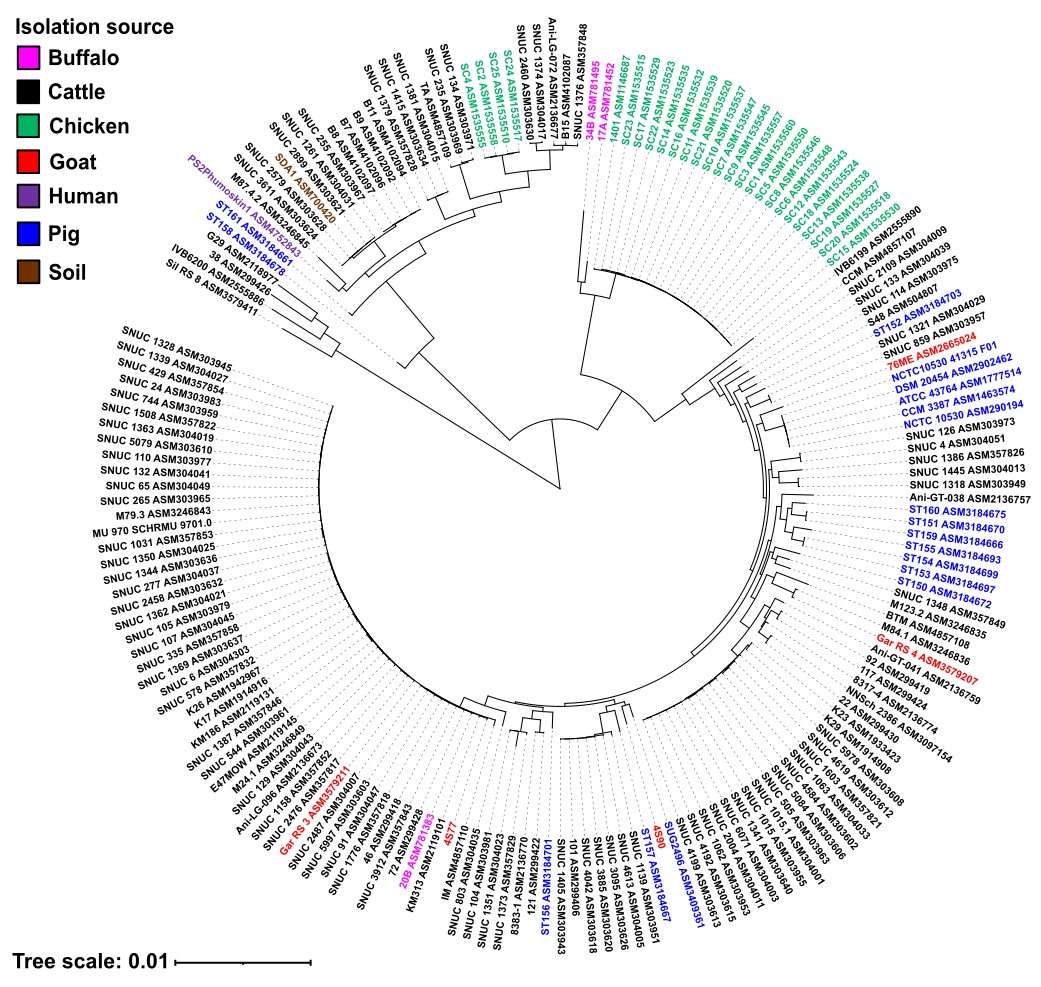


**Fig. S1.** **Core-genome phylogeny of 179 *Staphylococcus chromogenes* genomes.** The tree was constructed using single-nucleotide polymorphisms (SNPs) identified from conserved core regions, which covered approximately 65% of the reference genome (strain 17A). The dataset includes strains from diverse hosts and geographical locations, as listed in Table S2. Each label on the tree represents the isolate name followed by its corresponding assembly accession. ATCC 43764, CCM 3387, DSM 20454, and NCTC 10530 represent different culture collection designations of the same strain. Branch lengths represent the average number of nucleotide substitutions per site based on core-genome SNPs inferred by FastTree2, reflecting genetic distance among isolates (tree scale = 0.01).

**
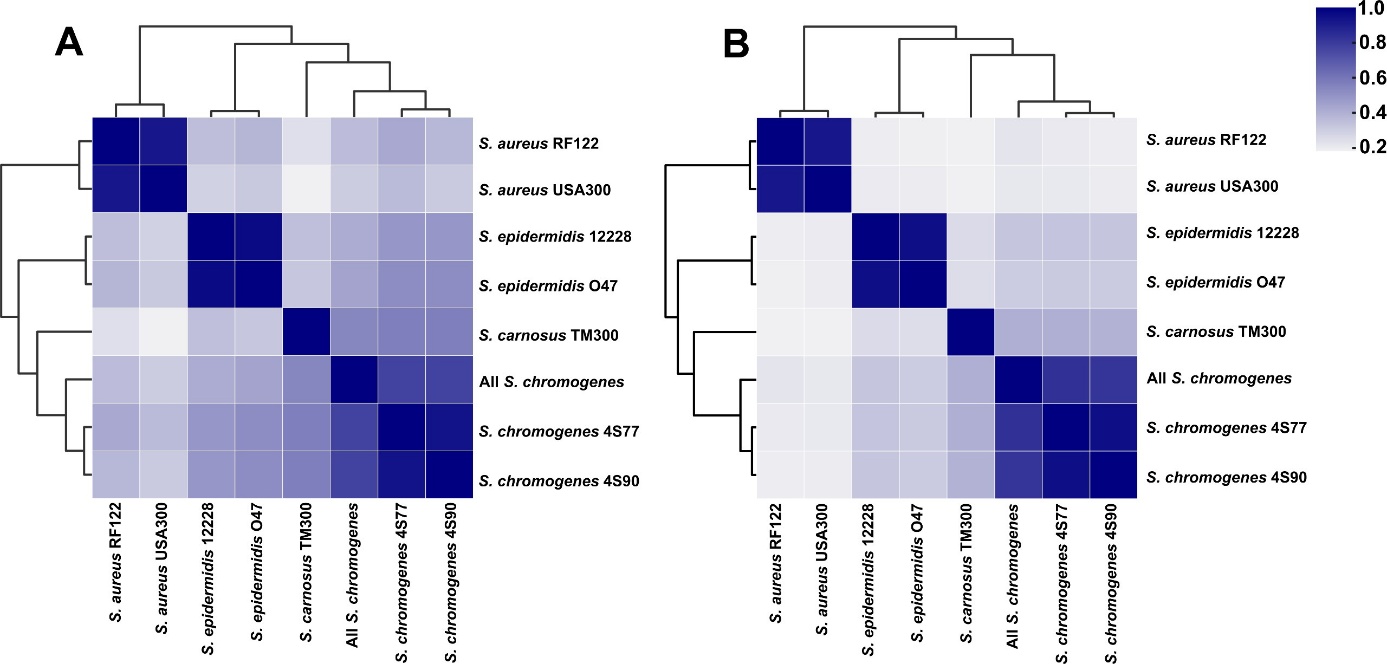
**

**Fig. S2. Pairwise Jaccard similarity matrices across different *Staphylococcus* genomes**. These matrices compare the genomes of two experimental *S. chromogenes* isolates (4S77 and 4S90), a composite group ("All *S. chromogenes*") representing six reference *S. chromogenes* genomes (17A, DSM 20454, 1401, IVB6199, IVB6200, and 76ME; accessions: GCA_007814525.1, GCA_029024625.1, GCA_011466875.1, GCA_025558905.1, GCA_025558865.1, GCA_026650245.1), and reference *Staphylococcus* strains representing a range of ecological and clinical backgrounds. These include *S. aureus* USA300_FPR3757 (Human skin and soft tissue infections, GCA_000013465.1), *S. aureus* RF122 (cattle mastitis, GCA_000009005.1), *S. epidermidis* O47 (nosocomial infections, GCA_013317125.1), *S. epidermidis* ATCC 12228 (non-pathogenic, GCA_000007645.1), and *S. carnosus* TM300 (non-pathogenic, GCF_000009405.1). (A) Jaccard similarity based on KEGG metabolic pathway presence/absence; (B) Jaccard similarity based on KEGG ortholog (KO) gene presence/absence. Similarity values range from 0 (no shared elements) to 1 (identical sets), with hierarchical clustering applied to both axes.

**
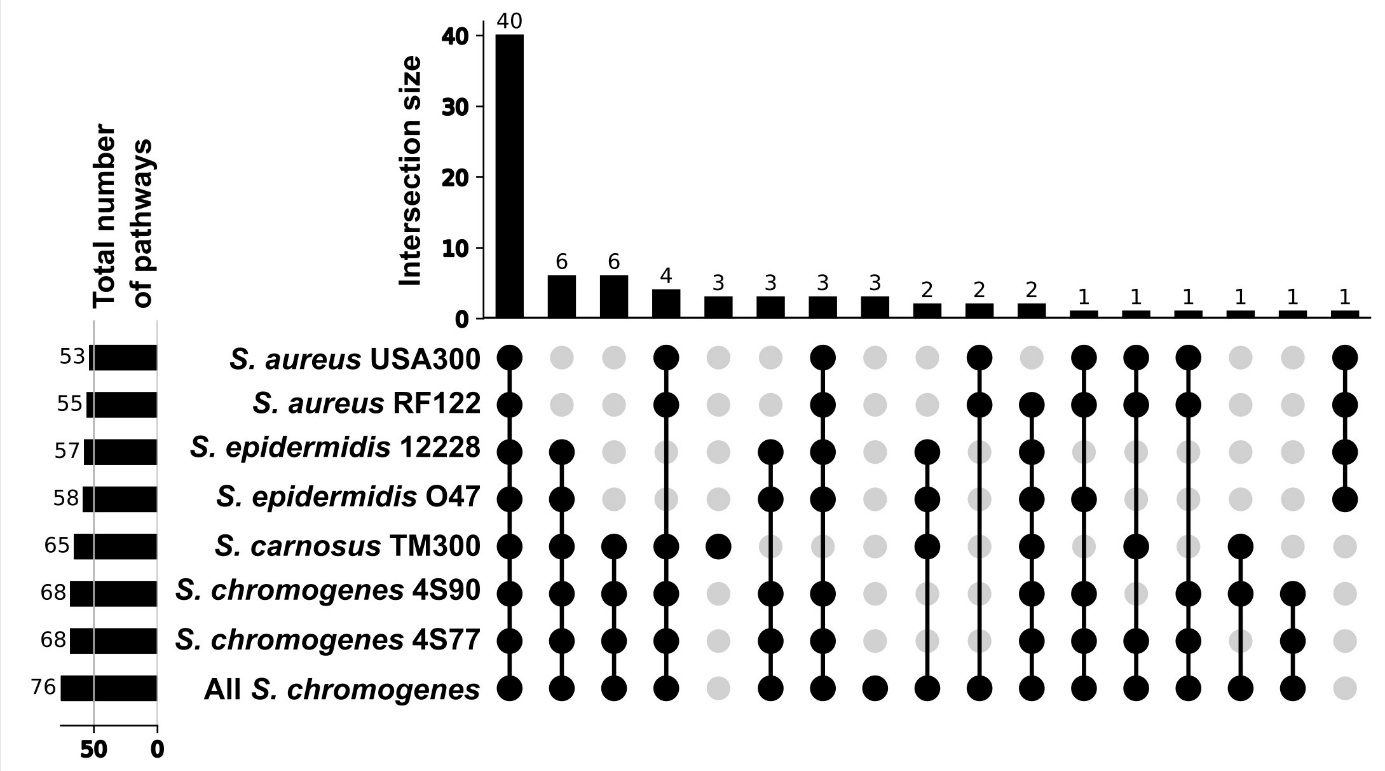
**

**Fig. S3. KEGG metabolic pathway overlap across *Staphylococcus* groups.** An UpSet plot showing the intersection of KEGG metabolic pathways across different *Staphylococcus* genomes, including two experimental *S. chromogenes* isolates (4S77 and 4S90), a composite group ("All *S. chromogenes*") representing six reference *S. chromogenes* genomes (17A, DSM 20454, 1401, IVB6199, IVB6200, and 76ME; accessions: GCA_007814525.1, GCA_029024625.1, GCA_011466875.1, GCA_025558905.1, GCA_025558865.1, GCA_026650245.1), and reference *Staphylococcus* strains representing a range of ecological and clinical backgrounds. These include *S. aureus* USA300_FPR3757 (Human skin and soft tissue infections, GCA_000013465.1), *S. aureus* RF122 (cattle mastitis, GCA_000009005.1), *S. epidermidis* O47 (nosocomial infections, GCA_013317125.1), *S. epidermidis* ATCC 12228 (non-pathogenic, GCA_000007645.1), and *S. carnosus* TM300 (non-pathogenic, GCF_000009405.1). Each vertical bar represents the number of KEGG pathways shared among the specific combination of groups indicated by the connected black dots beneath it. The horizontal bars on the left show the total number of pathways detected within each group. The largest intersection (n = 40) reflects pathways shared across all groups, while smaller intersections highlight pathways that are exclusive to one or several lineages.

**
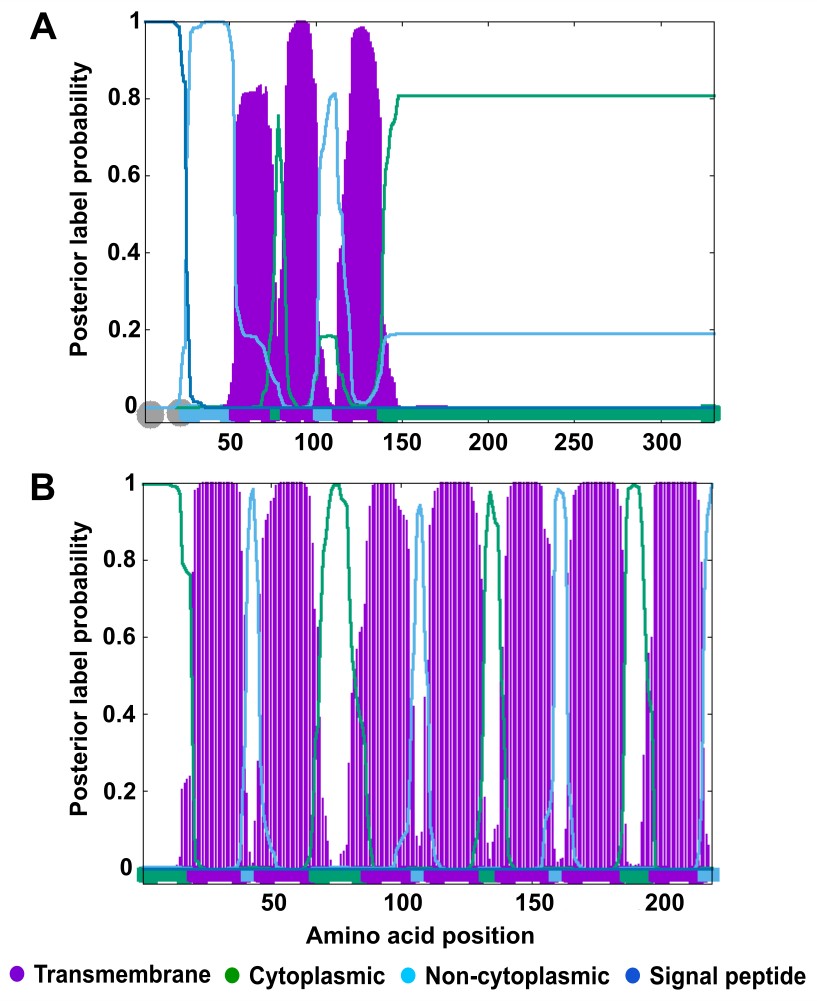
**

**Fig. S4.** **Phobius posterior probability plots for hemolysin III (Hly-III) family proteins.** (A) Predicted topology of the Hly-III family protein identified within the second prophage region of *Staphylococcus chromogenes* strain 4S90 (331 aa); (B) Predicted topology of the virulent Hly-III protein from *Bacillus cereus* (UniProt: P54176; 219 aa). The y-axis represents the posterior probability of each topological label assigned to the amino acid at the corresponding position along the x-axis. Higher values indicate greater confidence in the predicted topology for that region of the protein.

**
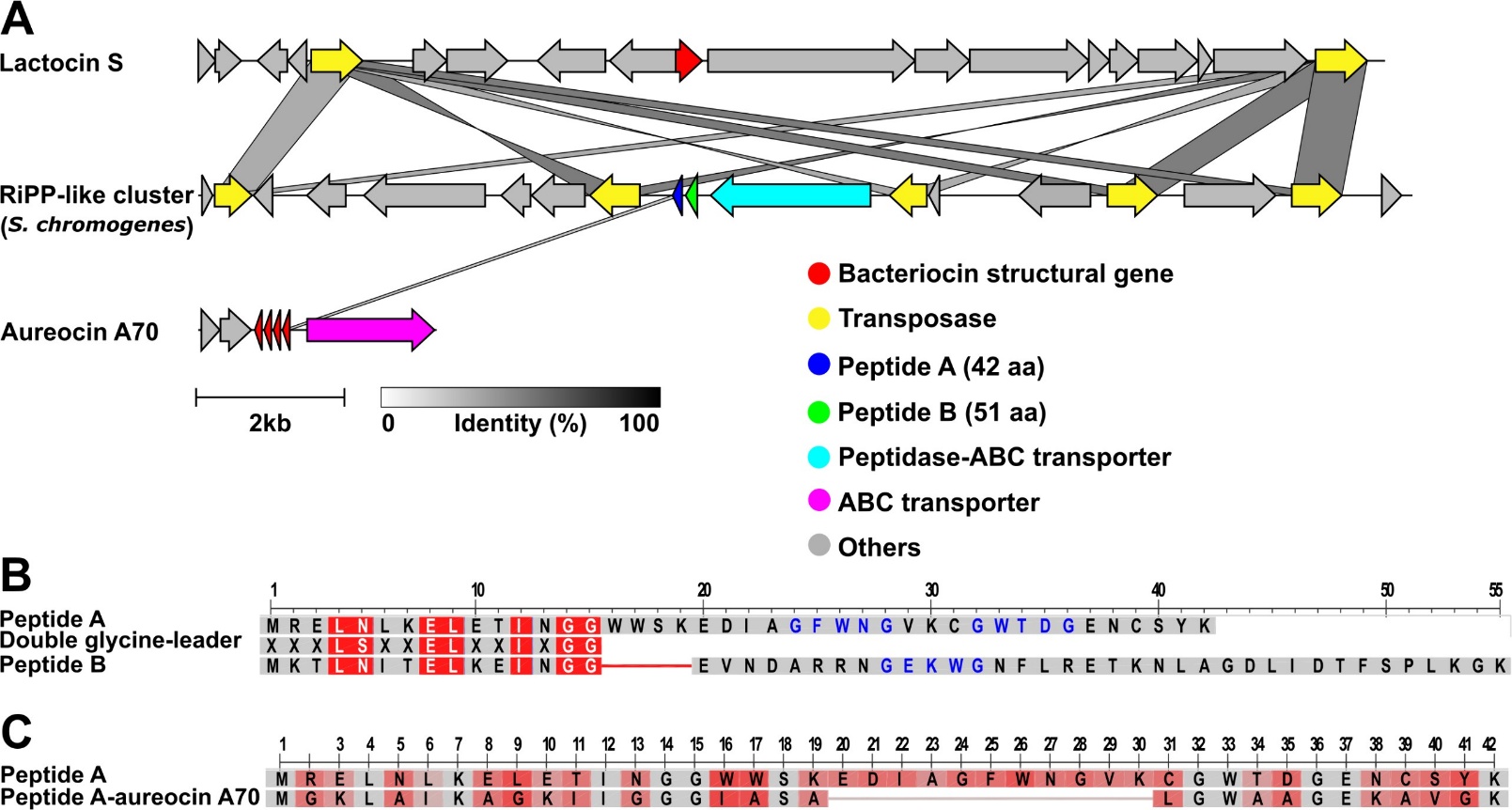
**

**Fig. S5.** **Comparative analysis of a RiPP-like bacteriocin gene cluster from *Staphylococcus chromogenes* strains 4S77 and 4S90.** The cluster is located between positions 12,816–24,975 on the 57-kb plasmid of strain 4S77 and 12,820–24,979 in 4S90. (A) Synteny analysis of the RiPP-like cluster located on the 57-kb plasmid of *S. chromogenes*, compared to the *lactocin S* cluster from *Lactobacillus sakei* (GenBank accession no. Z54312.2) and the *aureocin A70* operon (AF241888.2) from *S. aureus*. Arrows represent annotated genes, with colors indicating functional categories. Grey shading reflects sequence identity; (B) Alignment of peptides A (42 aa) and B (51 aa) from *S. chromogenes* with representative 15-residue N-terminal leader sequences from class IIb two-peptide bacteriocins, showing presence of the double-glycine leader. GxxxG motifs, another common feature of this class, are highlighted in blue; (C) Pairwise sequence alignment of peptide A from *S. chromogenes* and peptide A from aureocin A70, showing regions of identity and divergence. RiPP, ribosomally synthesized and post-translationally modified peptide.

**
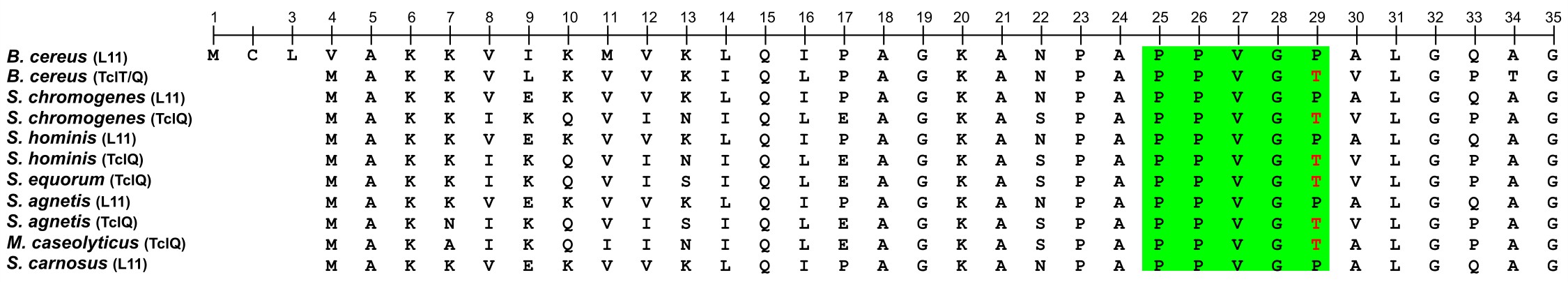
**

**Fig.** **S6. Multiple sequence alignment of the N-terminal region of 50S ribosomal protein L11.** The alignment includes the first 35 amino acid residues of ribosomal protein L11 from *Bacillus cereus* ATCC 14579 (GenBank accession no. QCX92219.1, QCX96850.1, QCX96853.1), *Staphylococcus chromogenes* 4S77 (FDAOAELG_02108, FDAOAELG_02353), *S. hominis* S34-1 (UJB23949.1, UJB24151.1), *S. equorum* KAVA (UVZ21322.1), *S. agnetis* 4244 (MBY7665565.1, MBY7665201.1), *Macrococcus caseolyticus* 115 (AIU53943.1), and *S. carnosus* TM300 (P36254.2). All strains except *S. carnosus* are known micrococcin P1 (MP1) producers. *S. carnosus* was included as a reference strain susceptible to MP1. For species with complete genomes, both the native L11 (*rplK*-encoded) and the alternative homolog encoded within the MP1 biosynthetic gene cluster (TClQ/T) are shown. For *S. equorum* and *M. caseolyticus*, only the cluster-encoded homolog was included due to incomplete genome data. The conserved proline-rich region in L11, shown in green, is where MP1 binds to block the ribosome. This region sits at the GTPase center, near 23S rRNA, and is crucial for proper function of the elongation factor G, which helps the ribosome move during protein synthesis. Substitutions of proline to threonine (shown in red) in the cluster-encoded L11 variants likely reduce MP1 binding, allowing strains to keep translating proteins.

**
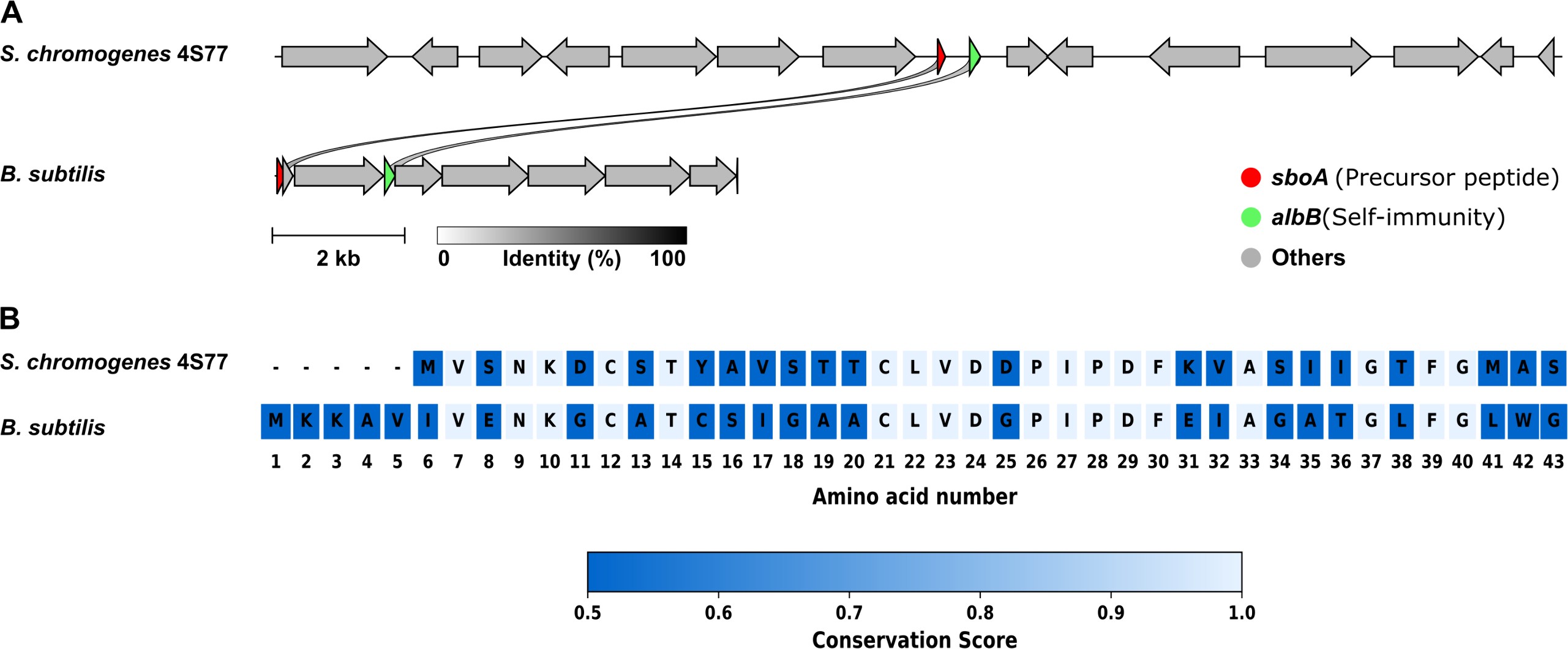
**

**Fig.** **S7. Subtilosin A-like gene cluster and precursor peptide alignment in *Staphylococcus chromogenes* 4S77.** (A) BAGEL4 analysis of the *S. chromogenes* 4S77 genome identified a subtilosin A-like operon on the chromosome (positions 235,620–254,823). The predicted cluster includes only two genes homologous to the subtilosin A operon from *Bacillus subtilis* 168 (NC_000964): a structural gene encoding the putative precursor peptide (*sbo*-like, FDAOAELG_00214; red) and a putative immunity gene (*albB*-like, FDAOAELG_00215; green). Other genes required for post-translational maturation (*albA*-*albG*) are absent. Shaded grey connectors indicate amino acid identity with homologous genes in the *B. subtilis* cluster; (B) Alignment of the predicted precursor peptide from *S. chromogenes* (FDAOAELG_00214) with subtilosin A from *B. subtilis* shows partial similarity in the core region. Overall similarity is visualised using a blue gradient scale.

**
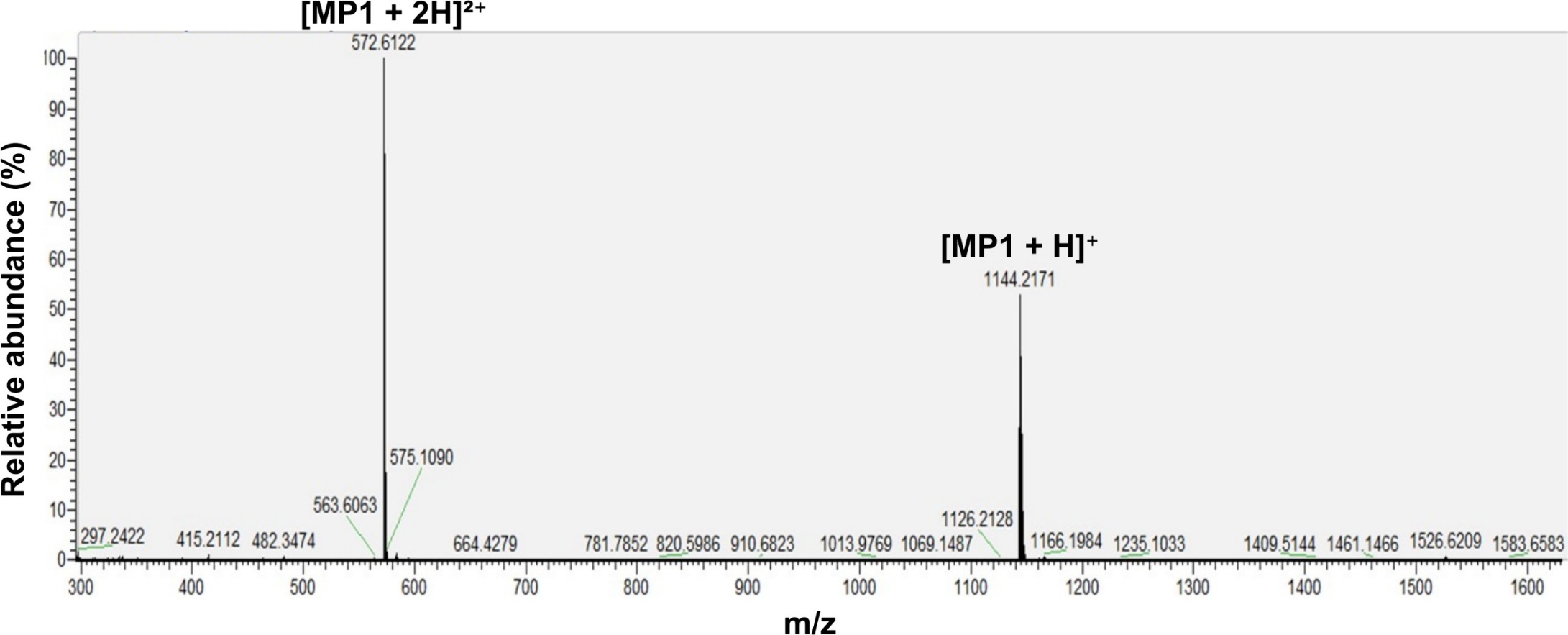
**

**Fig. S8. LC-MS analysis of the antimicrobial compound from *Staphylococcus chromogenes*.** The full-scan electrospray ionization mass spectrum shows a prominent base peak at mass-to-charge ratio (*m/z*) 572.6, corresponding to the doubly charged ion [MP1 + 2H]²⁺. A secondary peak at *m/z* 1144.2 corresponds to the singly charged ion [MP1 + H]⁺. The y-axis indicates relative abundance (%), with the most intense ion (*m/z* 572.6) normalized to 100%. These values are consistent with the expected molecular mass of micrococcin P1 (MP1).
