# Supplementary material for: Beyond commensalism: genomic insights into micrococcin P1-producing Staphylococcus chromogenes
Source: mSphere. 2025 Nov 24;10(12):e00733-25. doi: 10.1128/msphere.00733-25 (PMC12724294; doi:10.1128/msphere.00733-25)
Supplement: Supplemental Tables, part I — Tables S1, S4, S5, S9, and S13. [file msphere.00733-25-s0003.docx]

**Table S1.** General characteristics of *Staphylococcus chromogenes* strains with complete genomes retrieved from NCBI.

| **Strain** | **GenBank accession no.** | **Source** | **Host status** | **Chromosome size (bp)** | **GC content (%)** | **No. tRNA genes** | **No. rRNA genes** | **No. plasmid** | **Ref.** |
| --- | --- | --- | --- | --- | --- | --- | --- | --- | --- |
| 1401 | GCA_011466875.1 | Broiler bone | Lameness | 2,350,748 | 37 | 59 | 19 | 3 | [1] |
| 17A | GCA_007814525.1 | Buffalo milk | Healthy | 2,351,540 | 37 | 59 | 19 | 1 | [2] |
| 76ME | GCA_026650245.1 | Goat milk | Mastitis | 2,256,804 | 37 | 59 | 19 | 0 | [3] |
| BTM | GCA_048571085.1 | Cattle milk | Healthy | 2,273,649 | 37 | 59 | 19 | 1 | [4] |
| CCM | GCA_048571075.1 | Cattle milk | Healthy | 2,385,883 | 37 | 59 | 19 | 1 | [4] |
| DSM* | GCA_029024625.1 | Pig skin | NA | 2,280,008 | 37 | 59 | 19 | 1 | NA |
| IM | GCA_048571105.1 | Cattle milk | Intramammary infection | 2,342,667 | 37 | 59 | 19 | 2 | [4] |
| IVB6199 | GCA_025558905.1 | Cattle milk | Healthy | 2,264,062 | 37 | 60 | 19 | 0 | [5] |
| IVB6200 | GCA_025558865.1 | Cattle milk | Healthy | 2,371,745 | 37 | 59 | 19 | 0 | [5] |
| TA | GCA_048571095.1 | Cattle teat | Healthy | 2,372,466 | 37 | 59 | 19 | 1 | [4] |

 * DSM, *S. chromogenes* DSM 20454; NA, not available.

**References**

1. Ekesi NS, Hasan A, Parveen A, Shwani A, Rhoads DD. 2021. Embryo lethality assay as a tool for assessing virulence of isolates from bacterial chondronecrosis with osteomyelitis in broilers. Poult Sci.100:101455.

2. Pizauro LJ, de Almeida CC, Gohari IM, MacInnes JI, Zafalon LF, Kropinski AM, Varani AM. 2019. Complete genome sequences of 11 *Staphylococcus* sp. strains isolated from buffalo milk and milkers’ hands. Microbiol Resour Announc. 8:10.1128/mra. 01264-19.

3. Santos L, Nogueira J, Bezerra F, Novaes A, Gouveia J, Gouveia G, Da costa M. 2021. Sequencing, assembly and annotation of the genome of a *Staphylococcus chromogenes* isolate from goat mastitis.

4. Reydams H, Toledo-Silva B, Mertens K, Piepers S, Vereecke N, Souza FN, Haesebrouck F, De Vliegher S. 2024. Phenotypic and genotypic assessment of iron acquisition in diverse bovine-associated non-aureus staphylococcal strains. Vet Res. 55:6.

5. Akarsu H, Liljander A, Younan M, Brodard I, Overesch G, Glücks I, Labroussaa F, Kuhnert P, Perreten V, Monecke S. 2022. Genomic characterization and antimicrobial susceptibility of dromedary-associated *Staphylococcaceae* from the horn of Africa. Appl Environ Microbiol. 88:e01146-22.

**Table S4.** Isolated CRISPR elements in *Staphylococcus chromogenes* strains 4S77 and 4S90 identified by CRISPRCasFinder.

| **Strain** |  |  |  | **Direct repeat** | | | **Spacer** | |  |
| --- | --- | --- | --- | --- | --- | --- | --- | --- | --- |
|  | **Location** | **Start** | **End** | **Consensus** | **Length (bp)** | **No.** | **sequence** | **Length (bp)** | **Neighboring *cas* genes** |
| 4S77 | chromosome | 206600 | 206670 | ggtaattcatgagtgacacctgt | 23 | 1 | accgttaagtgaaatgtcgccttca | 25 | no |
| 4S77 | chromosome | 937152 | 937259 | gtgggttcgattcccatcagccgc | 24 | 1 | ccatatcgtaaaatgcgggtgtagtttaatggcaaaacctcagccttccaagctgatgtt | 60 | no |
| 4S77 | plasmid (~57 kb) | 1189 | 1305 | gttttttgttacatgtaacatgttac | 26 | 1 | atgttacctttattcttgttttttttcaacaactacttgttactctcttatcatatagctcaatc | 65 | no |
| 4S90 | chromosome | 211195 | 211269 | ttcagcataagcaggttcaccgtt | 24 | 1 | aataccgatgtgaccttctggtagtgg | 27 | no |
| 4S90 | chromosome | 941348 | 941455 | gtgggttcgattcccatcagccgc | 24 | 1 | ccatatcgtaaaatgcgggtgtagtttaatggcaaaacctcagccttccaagctgatgtt | 60 | no |
| 4S90 | plasmid (~57 kb) | 1189 | 1305 | gttttttgttacatgtaacatgttac | 26 | 1 | atgttacctttattcttgttttttttcaacaactacttgttactctcttatcatatagctcaatc | 65 | no |

**Table S5.** Isolated *cas* genes in *Staphylococcus chromogenes* strains 4S77 and 4S90 identified by CRISPRone.

| **Strain** | **location** | **Start** | **End** | **Gene** | **Type/Subtype** | **Strand** | **Product** |
| --- | --- | --- | --- | --- | --- | --- | --- |
| 4S77 | chromosome | 1296559 | 1296879 | mkCas0164:cas8a3 | I-A | + | Nucleotide pyrophosphohydrolase |
| 4S77 | chromosome | 1299214 | 1300185 | cd09655:casR | I | + | Biotin--[acetyl-CoA-carboxylase] ligase |
| 4S77 | chromosome | 1300205 | 1302889 | COG1199:DinG | IV-A | + | ATP-dependent helicase |
| 4S90 | chromosome | 1300524 | 1300844 | mkCas0164:cas8a3 | I-A | + | Nucleotide pyrophosphohydrolase |
| 4S90 | chromosome | 1303179 | 1304150 | cd09655:casR | I | + | Biotin--[acetyl-CoA-carboxylase] ligase |
| 4S90 | chromosome | 1304170 | 1306854 | COG1199:DinG | IV-A | + | ATP-dependent helicase |

**Table S9.** Putative virulence factors absent from *Staphylococcus chromogenes* 4S77 and 4S90.

| **Class** | **Factor** | **Gene(s)** |
| --- | --- | --- |
| Adherence | Accumulation associated protein | *aap* |
|  | Clumping factor A | *clfA* |
|  | Clumping factor B | *clfB* |
|  | Collagen adhesion | *cna* |
|  | Elastin binding protein | *ebp* |
|  | Cell wall associated fibronectin binding protein | *ebh, efb, uafA* |
|  | Extracellular adherence protein/MHC analogous protein | *eap/map* |
|  | Cell wall surface anchor family protein | *sasC, sasG, sraP* |
|  | Intercellular adhesin | *icaA, icaB, icaC, icaD, icaR* |
|  | Ser-Asp rich fibrinogen-binding proteins | *sdrC, sdrD, sdrE, sdrF, sdrG, sdrH, sdrI* |
| Exoenzymes | Cysteine protease (Staphopain) | *sspA, sspB, sspC, sspD, sspE, sspF* |
|  | Hyaluronate lyase | *hysA* |
|  | Serine protease | *splA, splB, splC, splD, splE, splF* |
|  | Staphylocoagulase | *coa* |
|  | Staphylokinase | *sak* |
| Host immune evasion | Capsular polysaccharide | *capA, capB, capC, capD, capE, capF, capG, capH, capI, capJ, capK, capL, capM* |
|  | Chemotaxis inhibitory protein of Staphylococcus | *chp* |
|  | Staphylococcal complement inhibitor | *scn* |
|  | Staphylococcal protein A | *spa* |
|  | Staphylococcal binder of immunoglobulin | *sbi* |
| Iron uptake and metabolism | Iron-regulated surface determinant | *isdA, isdB, isdC, isdD, isdE, isdF, isdG, isdH* |
|  | NPQTN-specific sortase B | *srtB* |
|  | Staphyloferrin B synthesis related | *sbnA, sbnB, sbnC, sbnD, sbnE, sbnF, sbnG, sbnH, sbnI* |
|  | Staphyloferrin A synthesis related | *sirA* |
| Toxins | Alpha hemolysin | *hly/hla* |
|  | Gamma hemolysin | *hlgA, hlgB, hlgC* |
|  | Leukocidin MI | *lukM, lukF-like* |
|  | Panton-Valentine leukocidin | *lukS-PV, lukF-PV* |
|  | Leukotoxin D | *lukD* |
|  | Leukotoxin E | *lukE* |
|  | Toxic shock syndrome toxin | *tsst* |
|  | Exfoliative toxin type A | *eta* |
|  | Exfoliative toxin type B | *etb* |
|  | Exfoliative toxin type C | *etc* |
|  | Exfoliative toxin type D | *etd* |
|  | Exfoliative toxin type I | *etI* |
|  | Type VII secretion system | *esaA, esaB, esaC, essA, essB, essC, esxA, esxB* |
|  | Phenol soluble modulins alpha | *PSMα1, PSMα2, PSMα3, PSMα4, PSMmec* |
|  | Phenol soluble modulins beta | *PSMβ1, PSMβ2, PSMβ3, PSMβ5, PSMβ6* |
|  | Enterotoxin A | *sea* |
|  | Enterotoxin B | *seb* |
|  | Enterotoxin C | *sec* |
|  | Enterotoxin D | *sed* |
|  | Enterotoxin E | *see* |
|  | Enterotoxin G | *seg* |
|  | Enterotoxin H | *seh* |
|  | Enterotoxin I | *sei* |
|  | Enterotoxin J | *sej* |
|  | Enterotoxin-like K | *selk* |
|  | Enterotoxin-like L | *sell* |
|  | Enterotoxin-like M | *selm* |
|  | Enterotoxin-like N | *seln* |
|  | Enterotoxin-like O | *selo* |
|  | Enterotoxin-like P | *selp* |
|  | Enterotoxin-like Q | *selq* |
|  | Enterotoxin-like R | *selr* |
|  | Enterotoxin-like U | *selu* |
|  | Enterotoxin-like V | *selV* |
|  | Enterotoxin Yent1 | *yent1* |
|  | Enterotoxin Yent2 | *yent2* |

**Table S13.** The plasmids in *Staphylococcus chromogenes* strains.

| **Strain** | **Plasmid** | **GenBank accession no.** | **Size (bp)** | **GC content (%)** | **Replicon type** | **No. AMR** | **AMR genes** | **No. VFs** | **VF genes** |
| --- | --- | --- | --- | --- | --- | --- | --- | --- | --- |
| 4S77 | unnamed1 | This study | 57,477 | 29 | rep7a | 0 | NA | 2 | *geh* |
| 4S90 | unnamed1 | This study | 57,607 | 29 | rep7a | 0 | NA | 2 | *geh* |
|  | unnamed2 | This study | 2,638 | 31 | rep21 | 1 | *lnuA* | 0 | NA |
| 1401 | unnamed1 | CP046029.1 | 80,178 | 31.5 | rep24c | 2 | *mphC, msrA, narA/B* | 0 | NA |
|  | unnamed2 | CP046030.1 | 43,387 | 35.0 | unknown | 0 | NA | 0 | NA |
|  | unnamed3 | CP046031.1 | 4,345 | 30.0 | rep7a | 0 | NA | 0 | NA |
| 17A | unnamed1 | CP031273.1 | 43,034 | 35.0 | unknown | 0 | NA | 0 | NA |
| BTM | pSCBTM | CP133243.1 | 22,957 | 30.0 | rep24a | 0 | NA | 2 | *geh, lip* |
| CCM | pSCCCM | CP133241.1 | 5,062 | 30.5 | rep21 | 2 | *lnuA* | 0 | NA |
| DSM* | unnamed1 | CP118954.1 | 20,779 | 29.0 | rep19c | 0 | NA | 0 | NA |
| IM | pSCIM1 | CP133245.1 | 24,411 | 29.5 | rep19c | 1 | *blaZ* | 1 | *lip* |
| IM | pSCIM2 | CP133246.1 | 4,397 | 31.5 | rep7a | 1 | *str* | 0 | NA |
| TA | pSCTA | CP133248.1 | 17,829 | 30 | rep19c | 0 | NA | 1 | *lip* |

* DSM, S. chromogenes DSM 20454. AMR, antimicrobial resistance, *blaZ*, beta-lactamase PC1; *geh*, glycerol ester hydrolase (lipase family); *lip*, lipase; *lnuA*, lincosamide nucleotidyltransferase; *mphC*, macrolide phosphotransferase; *msrA*, msr-type ABC-F proteins; NA, not applicable; *narA/B*, narasin resistance ATPase/permease; *str*, streptomycin resistance protein; VF, virulence factor.
